# Successful treatment with apixaban of sinus venous thrombosis due to pegylated asparaginase in a young adult with T cell acute lymphoblastic leukemia: case report and review of management

**DOI:** 10.1007/s00277-017-2930-0

**Published:** 2017-01-28

**Authors:** Laura Talamo, Michael Douvas, B. Gail Macik, David Ornan

**Affiliations:** 1 Department of Internal Medicine, Division of Hematology and Oncology, University of Virginia School of Medicine, PO Box 800716, Charlottesville, VA 22908-0716 USA; 2 Department of Radiology and Medical Imaging, University of Virginia School of Medicine, Charlottesville, VA USA

**Fig. 1:**
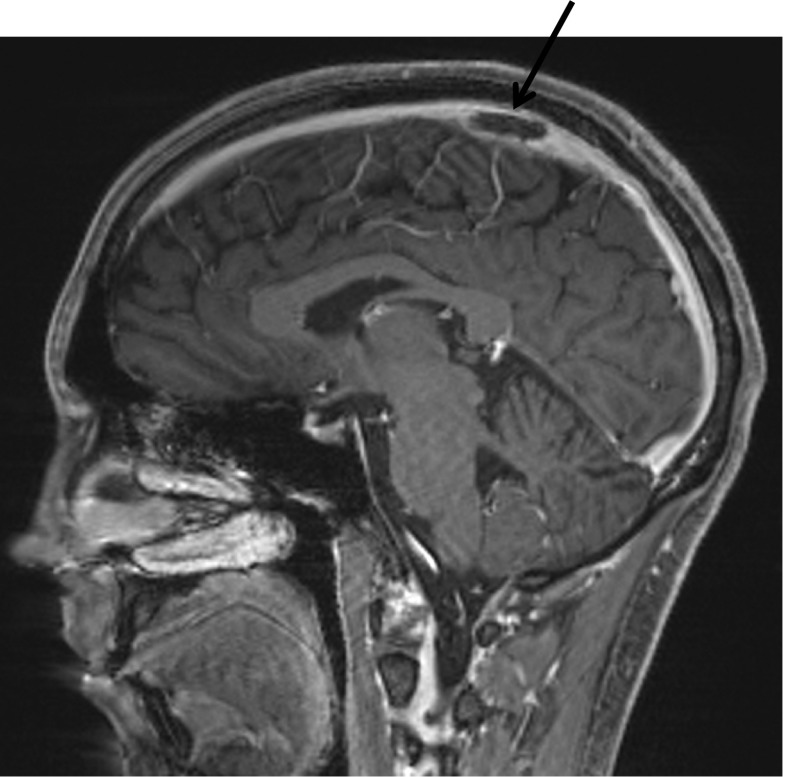
MRI demonstrating superior sagittal sinus thrombosis

Dear Editor,

A 22-year-old male presented in late 2015 with cough, fatigue, and weight loss. Chest x-ray showed large pericardial effusion, and chest CT confirmed the presence of a large anterior mediastinal mass (15.7 × 19.4 × 9.7 cm). Peripheral smear showed numerous blasts, and flow cytometry showed the blasts had a T cell immunophenotype with CD5, CD7, CD3, and TdT positivity. Bone marrow biopsy was consistent with T-lymphoblastic leukemia; microscopic examination of the core biopsy revealed a hypercellular marrow (approximately 90%) with predominantly blasts. Cytogenetic studies were normal. Treatment was initiated per augmented Berlin-Frankfurt-Munster (BFM) pediatric protocol, and he achieved remission after induction with subsequent negative testing for MRD. He received PEG-asparaginase as part of this regimen and after the third dose developed nausea and thumb tingling. His fibrinogen was low at 59 mg/dL (reference range 151–402 mg/dL), and he received one unit of cryoprecipitate. Two weeks later, he woke up with left arm numbness and tingling followed by a 2-min generalized tonic-clonic seizure and left-sided weakness. Brain MRI demonstrated superior sagittal sinus thrombosis with right superior frontal hemorrhagic venous infarct (Fig. 1). His fibrinogen was low at 77 mg/dL, and he received cryoprecipitate, which was followed by antithrombin (AT) concentrate (AT level 57%, reference range 83–128%) and then heparin infusion. Fibrinogen was checked twice a day with goal of >150 mg/dL. AT was checked every 12 h with a goal of 80%. The heparin infusion was continued for 3 days before transitioning to apixaban (10 mg twice a day to complete a 7-day load, followed by 5 mg bid).

**Fig. 2:**
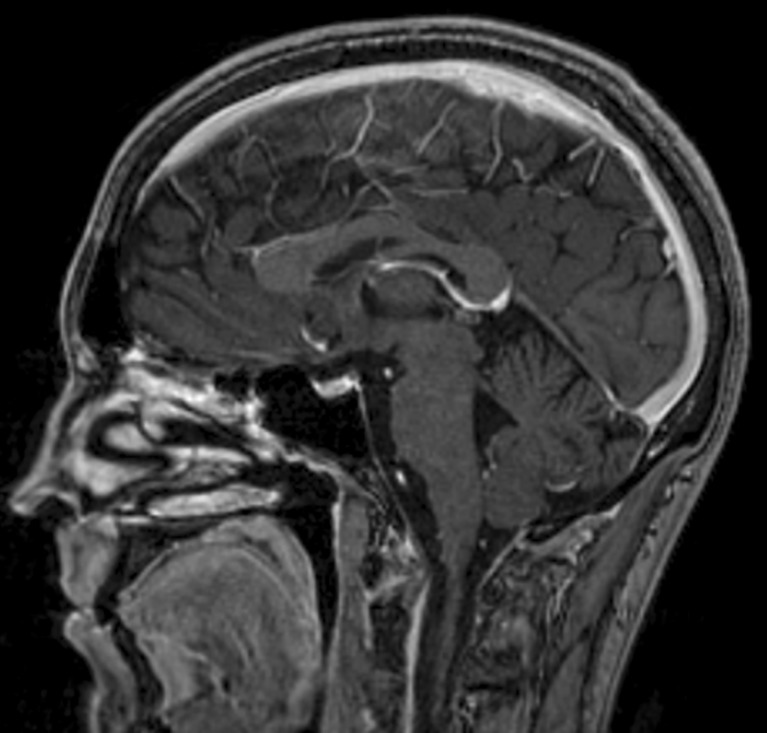
MRI after 2 months of treatment with apixaban, showing resolution of superior sagittal sinus thrombosis

MRI of the brain was repeated 2 months later and showed resolution of the parenchymal edema and the superior sagittal sinus thrombosis. No new dural venous sinus thrombosis was present (Fig. 2). Now, 9 months after his initial presentation, he remains on apixaban without any further neurologic episodes and no major or minor bleeding. He remains in remission and is being treated in maintenance.

The augmented BFM protocol has been utilized in the treatment of children with ALL [1] and has been shown to have efficacy in adults as well [2]. In fact, it has been shown to significantly improve outcomes in young adults such as our patient [3]. Asparaginase is a critical component of the regimen and improves outcomes [4–7]. Asparaginase impairs protein synthesis causing reduced plasma levels of coagulation factors fibrinogen, factor (F) V, FVII, FVIII, FIX, FX, FXI, and α_2_-antiplasmin [8]. This increased bleeding risk is balanced by impairment in the production of anticoagulant proteins antithrombin, protein C, protein S, and plasminogen. Fibrinogen and FVII recovery take place earlier than the recovery of the anticoagulant proteins, and one of the known toxicities of asparaginase is thrombus. The Dana-Farber Cancer Institute [9] found that of 548 patients with ALL treated with some form of asparaginase, and 9% of patients aged 20–30 years developed a venous thromboembolism (VTE). Two of the 18 adult patients who had VTE had sinus venous thrombosis.

Treatment of asparaginase-related VTE has historically been with heparin or low molecular weight heparin (LMWH). Our patient was treated with a heparin infusion for 3 days, before transitioning to the anti-factor Xa agent apixaban. Long-term recommendations for treatment include LMWH or warfarin. Given the efficacy of apixaban in treating venous thromboses and the low bleeding risk associated with its use when compared to warfarin [10, 11], we opted to treat our patient with apixaban rather than LMWH or warfarin. To our knowledge, this is the first report of the successful treatment with apixaban of a thrombosis associated with PEG-asparaginase administration. Our patient has had an excellent clinical outcome, with resolution of both his neurologic symptoms and venous thrombosis. Further testing of apixaban use in this setting is warranted.
